# Analyzing Sample Size and Cross-Contamination in Plastic Recycling: A Novel Thermal Analysis Method Coupled with Sampling Theory

**DOI:** 10.1021/acssuschemeng.5c01544

**Published:** 2025-05-07

**Authors:** Mahsa Akbari Lakeh, Floris Gerritsen, Tycho Aronds, Jeroen J. Jansen, Gerjen H. Tinnevelt

**Affiliations:** † Radboud University, Institute for Molecules and Materials (Analytical Chemistry), P.O. Box 9010, 6500 GL, Nijmegen, The Netherlands; ‡ Veridis Technologies B.V., High Tech Campus 27, 5656 AE Eindhoven, Netherlands

**Keywords:** plastic waste management, cross-contamination determination, differential scanning calorimetry (DSC), theory of sampling, polymer quantification

## Abstract

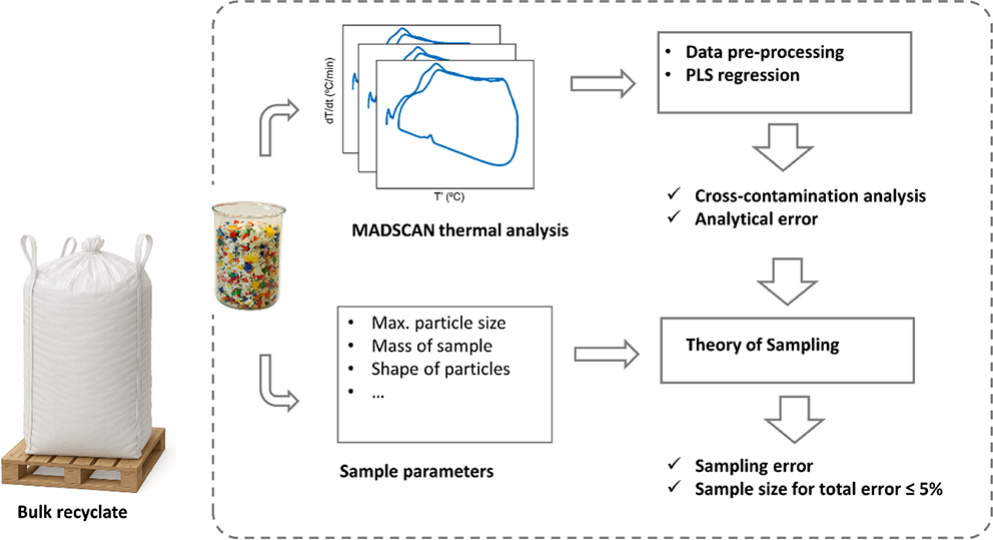

A crucial issue in the plastic recycling industry is the loss of quality in recycled materials due to cross-contamination, which leads to excessive material losses. Determining cross-contamination levels in recyclate batches fills a crucial gap in quality control, helping to identify suitable applications and enhance the value of the material stream. A key challenge lies in selecting a sample size that accurately represents the large variability within the tons of batches processed daily. This work presents a data analysis framework to accurately estimate cross-contamination levels in plastic recyclate batches and determine the sample size required to meet industry demands while accounting for both analytical and sampling errors. Additionally, this work introduces MADSCAN, a novel, scale-free thermal analysis technique that allows for the analysis of the sample sizes identified by the framework. Objectives include providing crucial information to industry stakeholders, assisting regulators in establishing quality control processes, and guiding technology providers in advancing measurement techniques for the circular economy, with a focus on meeting sample size and accuracy requirements.

## Abstract 2

This study advances plastic recycling sustainability by improving cross-contamination assessments and optimizing sample sizes for accurate quality control.

## 1 Introduction

A significant challenge in plastic recycling is the loss of quality in recycled materials. There is a notable discrepancy between the reported recycling rates and the proportion of high-quality plastic products manufactured from recycled materials, which is much lower. This indicates that much of the recycled plastic is used in applications where performance requirements specific to technical plastics are less critical. In Europe, only 13.5% of plastic products are made from recycled materials,^1^ with most being downcycled into low-value products. This highlights the common issue of quality loss in recycled materials, which reduces their economic value and restricts their applications.

The key factor contributing to this issue is cross-contamination,^2^ which occurs when different types of polymers are inadvertently mixed during the recycling process. This is a consequence of the inherent heterogeneity in plastic waste streams, which often contain diverse polymers alongside nonpolymeric contaminants. This mixing can result from inadequate separation during collection, processing, or sorting, as well as from process-related issues such as insufficient cleaning or mechanical blending.

Various sorting methods have been developed to minimize cross-contamination and enhance the purity of recycled streams.^3,4^ For example, optical sorting techniques, such as near-infrared (NIR) spectroscopy, are commonly employed for this purpose due to their speed and reliability in characterizing plastics. However, the high speeds necessary for handling large volumes of plastic waste make them particularly applicable at the sorting stage. Furthermore, the required sorting speed for economic viability may compromise sorting precision, particularly when dealing with multilayer materials, compounds (regranulate), contaminated surfaces, and black items.^5,6^ Therefore, complementary quality control (QC) techniques become essential in later stages, specifically after plastics are washed and shredded, to ensure that the final material meets quality requirements.

At the final stages of recycling, the washed flakes or granules need to be analyzed for cross-contamination levels to prevent quality loss. This analysis helps identify suitable applications for these recyclates as raw materials in the production of new plastic products. At this stage, speed is less critical than during bulk sorting, allowing for more detailed and accurate testing. Thermal analysis techniques, particularly Differential Scanning Calorimetry (DSC), are well-suited for identifying the composition of polymer blends.^7−9^ DSC is favored over techniques such as Nuclear Magnetic Resonance (NMR) for assessing cross-contamination because it requires no sample pretreatment in a laboratory environment. However, conventional DSC analysis, like most other techniques, typically involves sample sizes of only a few milligrams. Such small samples may fail to accurately represent the significant variability within large-scale material streams, where tons of plastic are processed daily.

Recent advancements in thermo-analytical technology have increased the capacity for sample mass,^10^ allowing for a more effective capture of sample heterogeneity. However, it is important to determine whether a single measurement from a fixed sample size can accurately represent the entire material stream. Therefore, it is essential to establish whether reliable predictions can be made to support evidence-based decisions regarding the material’s fate, considering both its value and composition.

The industry typically requires recyclate batches with a purity of 95% or higher and a maximum allowable total error of 5% (or 0.05 in fractional terms) for the compositional analysis of polymers. Since total error comprises both analytical and sampling errors, this highlights the importance of balancing cross-contamination levels, sample size requirements, and the precision and accuracy of measurements. Ensuring an adequate sample size is essential to avoid biased predictions from too small samples, allowing industries to assess the effectiveness of their current practices. The Theory of Sampling (TOS),^11,12^ developed by Pierre Gy in 1950, provides a comprehensive framework for optimizing sampling processes and determining sample sizes across various disciplines. By integrating technical and statistical principles, TOS ensures that collected samples and resulting calibration models are both reliable and representative.^13^ It addresses key aspects, such as estimating uncertainties from sampling operations and defining the sample size needed to achieve specific precision levels. Applying TOS principles helps to determine the minimum sample size required to meet the maximum allowable error limits in the recycling industry.

A comprehensive review^14^ of household waste studies examined waste components, various methods for analyzing waste composition, and sampling theory. This review highlights the lack of a universally adopted international standard for household solid waste studies and emphasizes the importance of determining sample sizes and addressing sampling errors according to TOS principles. In a related research, Maris et al. investigated the characterization of plastics from waste electrical and electronic equipment (WEEE) using mid-infrared (MIR) spectroscopy to develop a simplified methodology for recycling plants.^15^ Their study focused on methods for determining plastic composition in a 10-ton WEEE batch, emphasizing the importance of accurate sampling procedures, as outlined by TOS principles. While this work provided valuable insights into plastic characterization and recovery, the approach used to calculate sampling error and minimum sample size did not fully align with the original TOS principles and was therefore not adopted in the current work.

In this study, we present a novel data analysis framework for assessing the composition and cross-contamination levels of plastic recyclate batches, while determining the required sample size to meet industry demands. By applying MADSCAN, a scale-free thermal analysis technique, for the first time to analyze particulate plastic samples, we overcome the inherent limitations of conventional DSC methods, which rely on milligram-scale samples. Since polyolefins are among the most abundant plastics,^16,17^ this work specifically focuses on quantifying the cross-contamination of High-Density Polyethylene (HDPE) with polypropylene (PP) impurities, as well as Linear Low-Density Polyethylene (LLDPE) with Low-Density Polyethylene (LDPE).

Data analysis was conducted using the Partial Least Squares (PLS) method,^18,19^ building upon recent studies that demonstrated the efficacy of PLS models in estimating polymer blend compositions using conventional DSC measurements.^17^ In the proposed framework, the PLS model was validated using external samples from different industrial plastic waste streams, ensuring its applicability under real-world conditions. Crucially, we integrated the estimation of both analytical and sampling errors, using error propagation^20^ and Theory of Sampling (TOS) principles, to determine the minimum representative sample size necessary to achieve the industry-allowable maximum total error of 5%.

The proposed framework not only translates validated sample size measurements into evidence-based decisions but also advances the state-of-the-art quality control for plastic recycling. It provides regulators with actionable insights into future quality control requirements and helps technology providers refine measurement techniques to capture the full heterogeneity of industrial recyclate batches. Moreover, this approach bridges theoretical sampling principles with practical analytical challenges, thereby enhancing both the quality and economic value of recycled plastics and contributing to a circular economy.

## 2 Materials and Methods

### 2.1 Materials

This study investigates the cross-contamination levels of HDPE with PP impurities and LLDPE with LDPE in primary samples extracted from four distinct recyclate lots, each sourced from separate industrial waste streams. In the context of TOS, a *lot* refers to the target of sampling, encompassing all of the original material subject to sampling, such as a process stream, a stockpile, a barrel, or a lorry load. A *primary sample* is defined as the correctly extracted amount of material from the lot, with “correctly” meaning adherence to the principles and procedures outlined by TOS. The sampling characteristics of the lots under investigation are outlined in Table 1, providing insights into the composition variability of the plastic waste streams. Notably, in all cases, only one primary sample was available for each lot. From the primary samples, 20-g portions were extracted according to TOS guidelines and used as external validation (test) samples for MADSCAN analysis.

**Table 1:** Sampling Characteristics of the External Validation Samples

	Lot 1	Lot 2	Lot 3	Lot 4
Composition of lot	LDPE/LLDPE	LDPE/LLDPE	HDPE/PP	HDPE/PP
Polymer of interest, the analyte	LLDPE	LLDPE	HDPE	HDPE
Shape of particles	granule	granule	flakes	flakes
Mass of lot (g)	1.00 × 10^6^	1.00 × 10^6^	1.00 × 10^6^	1.00 × 10^6^
Average fraction of analyte in lot	0.70	0.70	0.95	0.97
Mass of primary sample (g)	6.80 × 10^2^	1.10 × 10^2^	6.00 × 10^2^	1.70 × 10^2^
Density of analyte in primary sample (g/cm^3^)	0.92	0.92	0.95	0.95
Density of matrix in primary sample (g/cm^3^)	0.92	0.92	0.91	0.91
Maximum particle size of analyte (cm)^a^	0.50	0.50	1.50	2.50
Particle size distribution^a^	0.55	0.55	0.25	0.25
Shape factor^a^	0.52	0.52	0.10	0.10

a Definitions and details for these parameters are provided in Section 2.4.3.

To estimate the cross-contamination levels and the minimum required sample sizes in each lot, two calibration sets were prepared. To ensure homogeneity and uniform composition across the samples, the calibration mixtures were prepared by using granules that were thoroughly mixed to minimize localized polymer aggregation. The LLDPE/LDPE calibration set was prepared by mixing granules at different ratios, as detailed in Table 2, ranging from 10 to 90 wt % of LLDPE. Pure samples of LLDPE and LDPE were also included in the calibration sets. Similarly, the HDPE/PP calibration set was prepared by mixing granules of these polymers at ratios specified in Table 2, and it incorporated pure PP and HDPE samples, resulting in a data set of ten samples.

**Table 2:** Composition of Calibration Sets for LLDPE/LDPE and HDPE/PP Mixtures

Calibration sets	Fraction of the polymer of interest (*) in calibration mixtures
LLDPE*/ LDPE	1.00, 0.90, 0.85, 0.80, 0.75, 0.70, 0.60, 0.40, 0.20, 0.10, 0.00
HDPE*/PP	1.00, 0.80, 0.70, 0.60, 0.50, 0.40, 0.30, 0.20, 0.10, 0.00

The calibration set materials were selected from well-defined commercial grades with known characteristics to ensure the reproducibility of the MADSCAN analysis. The external validation samples, obtained from recycling streams (Table 1), lack complete specification data. However, the calibration set, with its well-defined properties, provides a robust reference for validating the method. Table 3 summarizes the key specifications of the calibration materials, including the commercial name, density, melt flow rate, and relevant thermal and mechanical properties. Additional details can be accessed through the referenced sources.

**Table 3:** Specifications of Calibration Set Materials

Polymer	Commercial Name	Density	Melt Flow Rate	Key Properties
HDPE	Rigidex HD6070UA^21^	∼960 kg/m^3^	7.6 g/10 min (ISO 1133, 2.16 kg)	Melting Point: 132 °C; Tensile Yield: 31 MPa
LDPE	SABIC LDPE 2201H1^22^	∼922 kg/m^3^	0.85 dg/min (ISO 1133, 2.16 kg)	Designed for high-quality packaging films
LLDPE	ExxonMobil LLDPE LL 6101 Series Molding^23^	∼0.924 g/cm^3^	20 g/10 min	Peak Melting Temp: 122 °C; Tensile Yield: ∼ 10 MPa
PP	401-CB50^24^	Not reported	50 g/10 min (ISO 1133, 2.16 kg)	Tensile Yield: 24 MPa; Flexural Modulus: 1300 MPa

### 2.2 Data Collection

**Figure 1:**
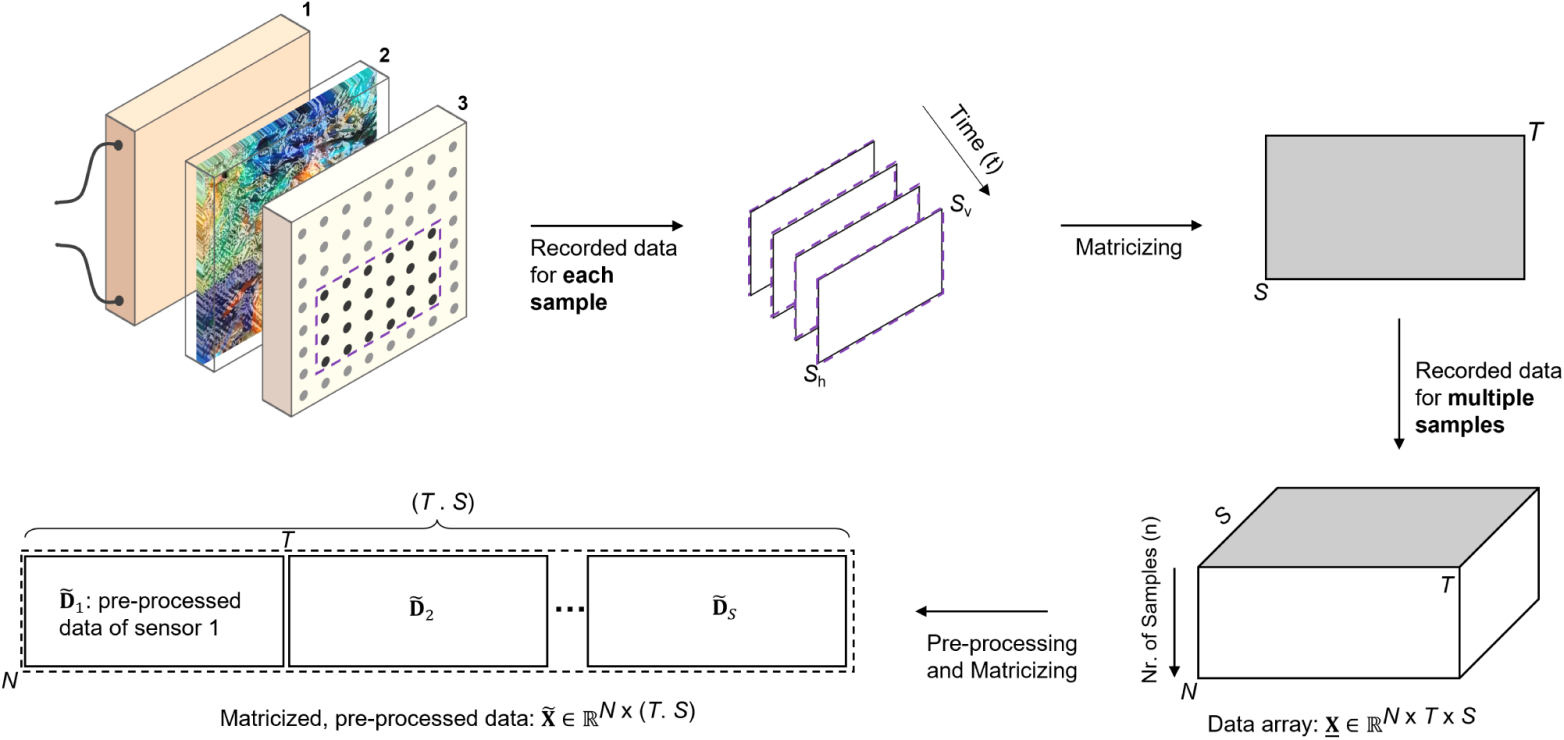
A schematic representation of the MADSCAN *T*-30 device, the generated data structure, and its preparation for further analysis. In the illustration of the device, sections 1, 2, and 3 correspond to the heating and cooling modules, the sample chamber, and the sensor array, respectively. These sections are in direct contact in practice. The sensor array is configured as *S*_h_ and *S*_v_ (with *S*_h_ = *S*_v_ = 8 in the present setup), where the sensors marked by black circles represent the subset (*S* = 4 × 6 = 24) analyzed in the current study. The three-way data recorded for a single sample and its matricization along the time dimension are shown at the top. The data array from multiple samples, **X**, shown at the bottom, is preprocessed and matricized into a larger matrix, , by concatenating the individual preprocessed sensor data matrices, , in the sample mode. Details on preprocessing steps are discussed further.

Thermal analysis was conducted on analytical samples using a MADSCAN *T*-30 device, which is currently an experimental laboratory instrument. The findings from this study will be used to translate this technology into an at-line deployable Process Analytical Technology (PAT). MADSCAN draws on the principles governing standard DSCs but allows for the analysis of much larger samples, enhancing the accuracy of quality control for sorted plastic materials. Unlike conventional DSC setups, MADSCAN features a vertical cuboid sample chamber with a narrow width, heated from one of its largest surfaces. Maintaining a fixed width while expanding other dimensions allows for larger sample volumes without increasing temperature differentials within the chamber. The vertical orientation of the chamber optimizes space utilization during sample melting, ensuring continuous contact between the plastic and both the heating and the sensor sides. The sensor side, opposite the heating element, has an 8 × 8 array of 64 sensors that monitor temperature variations. The heating/cooling layer is highly thermally conductive and in direct contact with the sample chamber, while the sensor array is made of a low thermal conductivity material to ensure localized temperature measurements. This configuration enhances the device’s ability to detect localized impurities, which traditional DSCs with smaller sample sizes cannot achieve. Figure 1 illustrates a schematic representation of the MADSCAN *T*-30 device and the data structure it generates.

For each experiment, a sample mass of 20.0 g was prepared and shaped by using a hot press to a thickness of 5 mm to ensure a precise fit within the sample chamber, thereby maintaining optimal thermal contact with both the heater and the sensor array. All measurements were conducted at room temperature. The thermal analysis protocol consisted of two heating cycles, from 50 to 350 °C, with a linear heating ramp of 5 °C/min. The cooling ramp was inactive, meaning that the device did not actively cool the sample back to the initial temperature between heating cycles. Each heating and cooling cycle lasted approximately 3.5 h, resulting in a total measurement time of about 7 h per sample. The first cycle was used to remove irregularities, while the second cycle produced cleaner peaks.

Future work will aim to reduce the total analysis time to 1–2 h by increasing the heating rate and implementing active cooling. These improvements in data acquisition are expected to enhance the analytical precision and better meet industrial quality control requirements in plastic recycling.

### 2.3 Data Preprocessing

**Figure 2:**
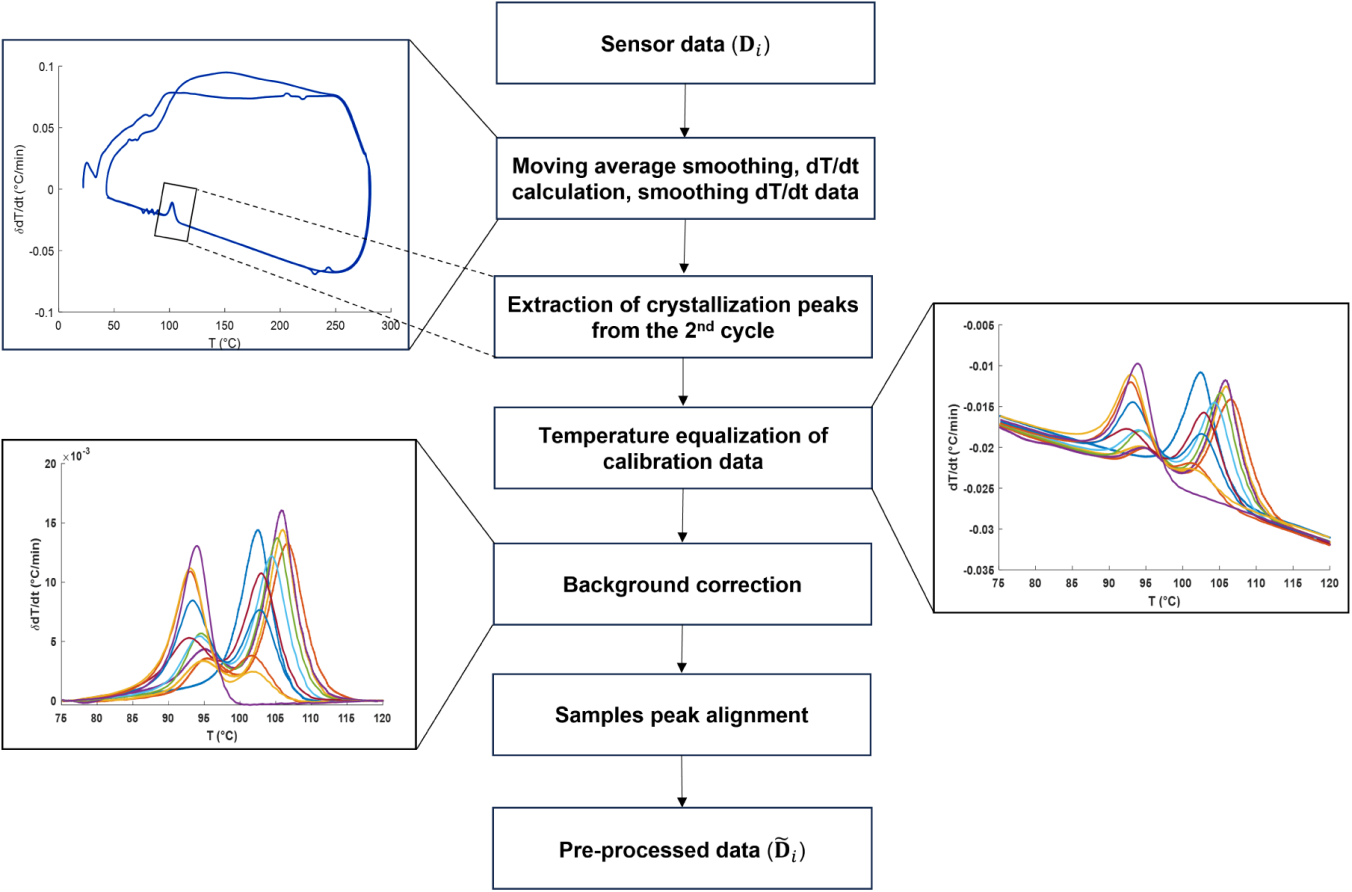
Overview of the applied workflow to process the MADSCAN data prior to multivariate analysis. Each preprocessing step was performed sequentially following the arrow direction. The inserted figures demonstrate the effect of each step on the data from mixtures of LLDPE–LDPE.

The first run of DSC removes irregularities caused by the material’s thermal history. As a result, the second run produces clearer and more consistent peaks, providing a more accurate representation of the material’s intrinsic thermal properties.^25^ Thus, for each composition of calibration sets and validation mixtures, the analysis was conducted on a second run. In these experiments, even without active cooling, the crystallization peaks remained clear and robust. In contrast, minor inconsistencies during the heating phase, arising from suboptimal heating control, resulted in melting peaks that were unclear or less reliable (see Figure 2, upper left panel). As a result, further analysis was focused on the crystallization peaks, which provide a more consistent signal sensitivity to variations in polymer composition. The raw data from the second cooling runs were smoothed by using a moving average filter. To better investigate the melting and crystallization features, the time derivative of the temperature values (d*T*/d*t*) was calculated and further smoothed to increase the signal-to-noise ratio. The crystallization peaks were then detected using the approach proposed in ref. (17) A temperature window was selected that covered the entirety of the crystallization peaks while extending slightly beyond their boundaries to incorporate representative segments of the baseline for further correction. Careful consideration was given to avoid including extraneous noise or unrelated/unexpected peaks.

Multivariate calibration requires standardization of the temperature profiles of different samples scanned at varying resolutions in DSC experiments. For this purpose, the temperature profiles were binned into predefined intervals, and the mean temperature within each interval was used. As depicted in Figure 2, the crystallization peaks were superimposed on a drifting baseline that could obscure the true thermal events of interest in the DSC data. For baseline correction, the peaks within the selected temperature range were removed, and the isolated baseline component was fitted to a second-order polynomial. The selection of the polynomial order was determined by exploring polynomial functions ranging from first to fourth order. The second-order polynomial yielded the most optimal results in terms of accuracy and precision for the peaks investigated. Subsequently, the fitted baseline was subtracted from the d*T*/d*t* data.

Two types of shifts were observed in the data: the shifts in crystallization peaks among different compositional samples measured on the same sensor and the shifts in peaks among different sensors. While the former shifts were relatively smaller and manageable, the latter shifts were more severe and sample-dependent. Consequently, peak alignment was performed only on different samples of each sensor using the correlation-optimized warping (COW) approach.^26^ Peak alignment with COW involved optimizing two key parameters, the segment length and slack size, for data from each sensor. In addition, the selection of the reference vector for the COW procedure was guided by the maximum cumulative product of correlation coefficients criterion. The issues related to the peak shifts are caused by the current MADSCAN setup and will be addressed in future versions. Various preprocessing steps applied to the MADSCAN profiles of each sensor are summarized in Figure 2.

### 2.4 Statistical Analysis

#### 2.4.1 Partial Least-Squares Regression (PLSR) Analysis

Partial least-squares regression (PLSR) was used to model and predict variations in polymer composition (wt % of LLDPE, LDPE, HDPE, and PP) based on their respective MADSCAN profiles. PLSR, which is a widely used multivariate calibration method,^18,19^ can address the collinearity inherent in DSC profiles by projecting data into a lower-dimensional space. All MADSCAN profiles from the calibration sets listed in Table 2 were utilized to construct the calibration models. Two approaches were examined: the first involved building individual PLSR models for each sensor’s data, denoted as  in Figures 1 and 2, and the second used a single PLSR analysis on the matricized data set  formed by row-wise concatenation of the individual sensor matrices. Figure 1 illustrates both the data structure generated by the MADSCAN *T*-30 setup and the matricization approach. This approach integrates diverse sensor outputs while preserving time-dependent variations, thus enabling the model to benefit from the combined information despite the challenges in correcting peak misalignments among sensors (see the Data Preprocessing section). Although deconvolution-based methods could also be applied, their expected calibration performance is, in this case, comparable to that obtained with PLSR. Consequently, PLSR was selected for its reliability and computational efficiency. Model performance was evaluated by using leave-one-out cross-validation (LOOCV) to determine the fractions of LLDPE and HDPE in the first and second calibration sets, respectively. From the individual PLSR models, the average root-mean-square error of cross-validation (RMSECV) and the average R^2^ values for both calibration and cross-validation were calculated. For external validation, analytical samples from the investigated lots were examined. Finally, the uncertainty of the results was investigated by estimating analytical and sampling errors by using the methods discussed further.

#### 2.4.2 Calculation of Total Analytical Error (TAE)

Total analytical error (*S*_*TAE*_) represents the deviation of an individual result from the reference concentration of the analyte in the sample and thus encompasses both the systematic and random components of the error simultaneously.^27^ There is a direct mathematical link between TAE and measurement uncertainty.^28^ If all sources of systematic error or bias are presumed to be eliminated or corrected, uncertainty would serve as an adequate measure of the analytical results’ reliability.^27^ Resampling and error propagation are two primary approaches for estimating the uncertainty of analytical measurements in multivariate analysis.^29^ The advantage of the latter is its ability to generate closed-form expressions to provide the relative impact of the different sources of uncertainty on the resulting prediction errors.^30,31^ In this work, eq 1 is used to estimate the propagation of homoscedastic and uncorrelated noise into the composition values:^30,31^
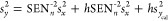
1

Here, SEN*_n_* (in signal × concentration^–1^ units) denotes the sensitivity of the *n*th component of interest in a multicomponent sample;  (in signal^2^ units) defines the variance in instrumental signals; *h* (dimensionless) indicates the sample leverage, which measures the position of the sample relative to the calibration space; and  (in concentration^2^ units) denotes the variance in calibration concentrations. The three terms of this equation address the propagation of uncertainty from instrumental signals in the test sample data, instrumental signals in the calibration data, and calibration concentrations, respectively.

As shown in eq 2, sensitivity plays a crucial role in evaluating the uncertainty of the analytical results. Sensitivity is analyte-specific and is influenced by both the signal type and the algorithm used in constructing a calibration model. Olivieri and his coworkers introduced a general equation for determining sensitivity that employs uncertainty propagation.^20,32,33^ When first-order calibration was applied using PLS, sensitivity can be expressed as follows:
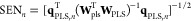
2
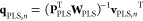
3
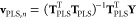
4

where **v**_PLS,*n*_ (defined in eq 4) is the vector of PLS regression coefficients in latent space, **W**_PLS_ is the matrix of PLS calibration weights, and **P**_PLS_ is the loading matrix of predictor variables. **T**_PLS_ is the score matrix of predictor variables, and **Y** is the matrix of target variables (e.g., concentration).

#### 2.4.3 Estimation of Total Sampling Error (TSE)

Based on the theory of sampling,^13^ total sampling error (*S*_*TSE*_) arises from both the material properties, particularly their heterogeneity, and the sampling process. Therefore, improving results requires adopting both a proper sampling process and a well-defined sampling plan.^13^ When all sources of sampling error are eliminated through appropriate sampling processes and plans, the fundamental sampling error (FSE) represents the practical minimum sampling error. Estimating the FSE is useful for assessing and optimizing all sampling steps, particularly when thorough mixing prior to sample extraction is feasible. The FSE is inherent to the lot’s heterogeneity and material properties, such as the shape, size, density, and composition of particles. Gy’s formula, shown in eq 5, was used to approximate the relative variance of the FSE, a commonly applied method for practical purposes:
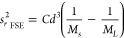
5
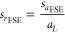
6

where *S*_*r FSE*_ is the relative standard deviation, and *S*_*a FSE*_ (in concentration units) is the absolute standard deviation of the FSE; *a*_*L*_ is the average concentration of the component of interest in the lot; d is the characteristic particle size (in cm), defined as the opening of a square mesh that retains no more than 5% of the particles (refer to the maximum particle size of the analyte in Table 1); *M*_*S*_ is the sample size (in g); *M*_*L*_ is the lot size (in g); and *C* is the “sampling constant,” which depends on the properties of the material sampled. The sampling constant is the product of four material parameters that are listed in eq 7.
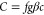
7

Here, *f* represents the ″particle shape factor″ (dimensionless), defined as the ratio of the volume of sampled particles with dimension *d* to the volume of a cube of the same dimension. For particles shaped like a cube, *f* = 1; for sphere-shaped particles, *f* = 0.52; and for nearly flat disc particles or flakes, *f* = 0.1. The parameter *g* is the “size distribution factor” (dimensionless) describing the span of the particle sizes in the lot. For wide size distribution and noncalibrated materials *g* = 0.25; for calibrated materials (two consecutive sieve openings of a certain series) *g* = 0.55; and for naturally calibrated materials, such as cereals *g* = 0.75. β is the “liberation factor” (dimensionless) describing the liberation of the critical component from the matrix. β varies between 0 and 1, where *β*_max_ = 1 for fully liberated particles and *β*_min_ = 0 for totally incorporated particles. The final parameter, *c*, is the “constitution factor” (g/cm^3^), which quantifies the contribution of particle density variability to the heterogeneity of a sample. It reflects the density contrast between the constituent of interest and the surrounding matrix, thereby influencing the fundamental sampling error. The constitution factor can be estimated using the following formula:
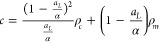
8

Here, α and *ρ*_*c*_ represent the concentration and density of the constituent of interest, respectively, while *ρ*_*m*_ indicates the density of the matrix. For materials consisting of fully liberated pure components, such as physical mixtures of different polymers examined in this study, the formula simplifies to eq 9:^12^
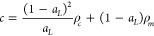
9

In this simplified form, *c* represents an upper limit of the density variability’s effect on sample heterogeneity. Note that although *a*_*L*_ is often given as a percentage, it must be expressed as a fraction (i.e., between 0 and 1) for these calculations.

#### 2.4.4 Total Error (TE) and Minimum Sample Size Determination

The total error associated with an analytical result comprises both the total analytical error (TAE) and the total sampling error (TSE).^34^ Since the variances of these errors are additive, the total error can be estimated as follows:
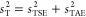
10

Here, *S*_T_, *S*_TSE_, and *S*_TAE_ represent the standard deviation of the total error, total sampling error, and total analytical error, respectively.

The FSE model can be used to estimate both the variance of a given sampling step and the minimum sample size *M*_*S*_ for a fixed uncertainty level, . If the total error for an experiment is fixed and the analytical error of the measurements is known, then the uncertainty of the FSE can be determined. When the lot mass is significantly greater than the sample size, a simplified formula can be used for determining the minimum sample size:
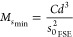
11

By using eq 8 to calculate the constitution factor, the original Gy’s formula can be effectively applied to estimate the fundamental sampling error and the minimum sample size for physical mixtures of polymers. This approach is preferable to the method used in ref. (15), which produced unsatisfactory results in our analysis.

#### 2.4.5 Software

Data analysis was conducted using MATLAB 2021a software (MathWorks Inc., Natick, MA, USA). The figures of merit for the PLS models were calculated using MVC1 (Multivariate Calibration 1) codes available in MATLAB.^35^

## 3 Results and Discussion

This study introduces a data analysis framework that leverages PLS analysis to estimate cross-contamination levels in plastic recyclate batches while evaluating both sampling and analytical errors. MADSCAN technology was employed because it allows for the acquisition of larger sample sizes compared with conventional techniques, thereby providing a more accurate estimation of batch heterogeneity. The current MADSCAN setup uses a maximum sample size of 30 g, and the framework assesses whether this configuration meets the industrial requirement of a maximum allowable total error of 5%. The following sections demonstrate the efficacy of the MADSCAN technique, coupled with PLS analysis, in accurately determining the compositions of HDPE and LLDPE in industrial lots. Additionally, the minimum sample size required to meet industry-acceptable error limits (≤5%) is determined based on sampling theory, and the sample size needed to achieve even lower error thresholds (e.g., 1% or 0.01 in fractional terms) for regulatory decisions is also explored.

### 3.1 Data Preprocessing Effects

In the examination of the sensor signals, inconsistencies were observed in the data recorded from some sensors, including absent or unclear transition peaks and, occasionally, unexpected noisy peaks. These challenges were attributed to edge effects and incomplete sample chamber coverage, both of which are linked to the geometry of the sample chamber. Edge effects refer to variations in heat transfer and airflow that mostly occur near the edges of the sample chamber, impacting the quality of the data. Incomplete sample chamber coverage typically occurs due to the lowering of the sample level resulting from melting and subsequent crystallization. Addressing these effects is necessary to ensure accurate and consistent analysis. The study revealed that 24 of the 64 available sensors, arranged in a 4-by-6 grid, provided consistent and reliable data. These sensors, marked with black circles in Figure 1, were selected for further analysis.

**Figure 3:**
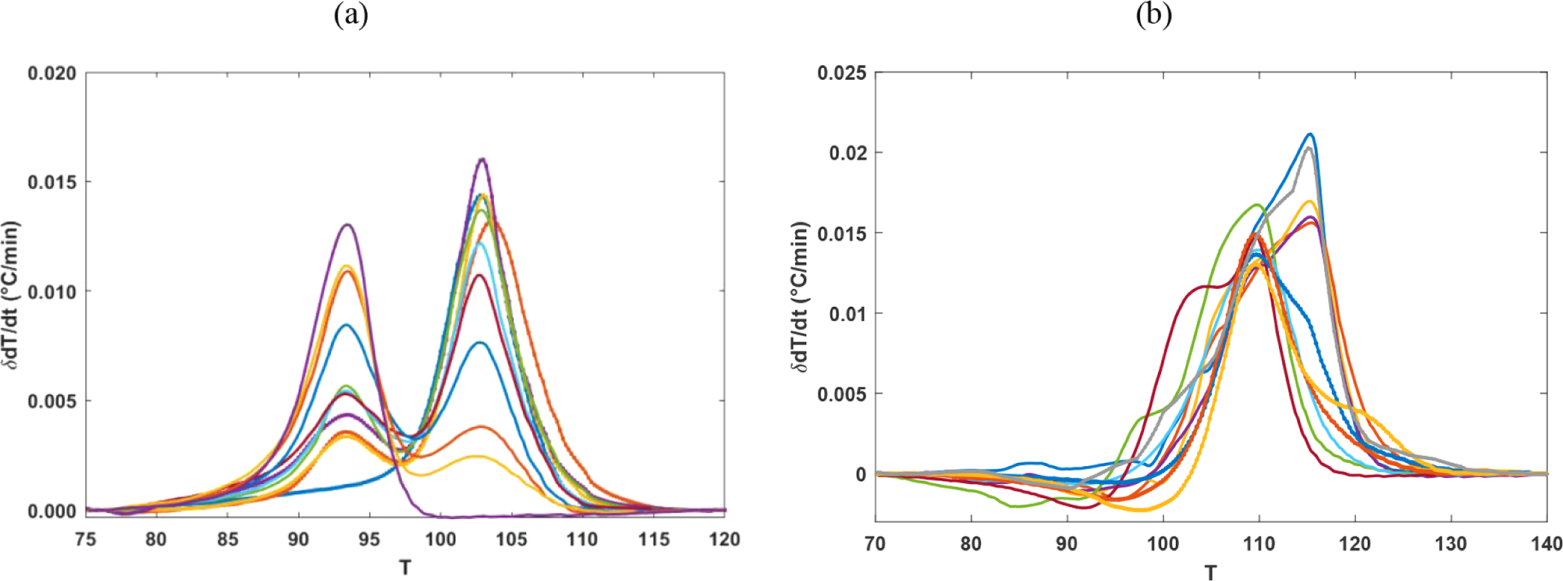
MADSCAN data from a randomly selected sensor in (a) LLDPE–LDPE, and (b) PP–HDPE calibration sets after preprocessing.

Following the extraction of the smoothed, temperature-equalized crystallization peaks, background correction was applied to each MADSCAN profile. Subsequently, peak alignment was performed individually for the calibration data set of each sensor. Figure 3 shows the preprocessed data from a randomly selected sensor within the LLDPE–LDPE and PP–HDPE calibration sets. The crystallization peak positions, shown in Figure 3, deviate from the expected temperature ranges due to sensor-specific shifts. While these data are valuable for quantitative analysis, they are not intended for the qualitative identification of polymer types in mixtures. Instead, the objectives of the PLS analysis are to quantify the fraction of polymers of interest and provide an estimate of the analytical error in this quantification.

### 3.2 PLSR Analysis

Table 4 summarizes the calibration, cross-validation, and prediction parameters of the PLS models for the desired polymers. For LDPE–LLDPE mixtures, the accuracy of both approaches was comparable. However, the standard deviation of the matricizing approach was significantly lower, approximately one-third of the average standard deviation of the individual models (0.006 versus 0.018), indicating higher precision. For PP-HDPE mixtures, the overall precision was comparatively lower, with the standard deviation of the individual models’ data being approximately 1.5 times that of the matricized data (0.039 versus 0.026). Hence, the precision of the models constructed using matricized data was higher compared to the average precision achieved with individual models. The observed increase in precision of models built on matricized data can be attributed to several factors. By combining data sets from multiple sensors into a single, unified data set, the model benefits from the integration of diverse information, capturing a broader range of data characteristics and intersensor correlations, leading to more comprehensive and reliable predictions. This approach effectively averages out the errors from individual sensor data, reducing the overall error propagation. Additionally, the matricizing approach takes advantage of the interdependencies and shared patterns among the sensors, which enhances the model’s predictive accuracy. This method not only simplifies the modeling process but also ensures consistent and robust quantification of analytes across different sensor inputs, ultimately resulting in higher precision.

**Table 4:** Regression, Validation, and Prediction Parameters of PLSR Models

Analyte	Model type	RMSECV	R^2^ Cal.	R^2^ CV	Predicted fraction ± SD.
LLDPE	Individual models	0.06	0.99	0.96	0.69 ± 0.02
	Matricized data model	0.04	1.00	0.99	0.70 ± 0.01
HDPE	Individual models	0.13	0.93	0.81	0.88 ± 0.04
	Matricized data model	0.10	0.97	0.88	0.89 ± 0.03

### 3.3 Sampling Error and Minimum Sample Size Requirements Based on TOS

The estimated standard deviations of the matricized data models were used as an estimate of the analytical uncertainty in quantifying the LLDPE and HDPE fractions. It was assumed that the analytical error for the samples from lots 1 and 2, as well as those from lots 3 and 4, was the same because of the identical composition of the first two lots and the closely resembling compositions of lots 3 and 4. In the subsequent step, the fundamental sampling error (FSE) was estimated for each lot based on the properties of particles using eq 5. All components of the total sampling error, except for FSE, were expected to be negligible by adhering to the correct sampling guidelines outlined in TOS. The constitution factor for each lot was computed using eq 9. As Table 5 indicates, the lowest constitution factor was found in the fourth lot due to its purity, as it contains 97 wt % HDPE. Gy’s formula, detailed in Section 2.4.3, was employed to estimate the standard deviation of the FSE (see Table 5). In addition to the FSEs, the corresponding coefficients of variation (CV) were calculated to facilitate a comparison of sampling errors across different lots. Although the differences in sampling errors between lots are generally small when considering the size of the primary samples, this error is highest in lots 2 and 4, which can be attributed to their smaller primary sample sizes. Notably, the highest CV was observed in lot 2, which had the smallest primary sample size.

**Table 5:** Values of Constitution Factor and Fundamental Sampling Error Calculated for Each Lot

Lots	Constitution factor (g/cm^3^)	Variance of relative FSE	FSE (polymer fraction)	CV (%)^a^
Lot 1	0.39	2.07 × 10^^-^5^	0.0032	0.46
Lot 2	0.39	1.28 × 10^^-^4^	0.0079	1.13
Lot 3	0.05	6.75 × 10^^-^6^	0.0025	0.26
Lot 4	0.03	6.47 × 10^^-^5^	0.0078	0.80

a Coefficient of variation (CV) is defined as the square root of the variance of the relative FSE multiplied by 100.

**Figure 4:**
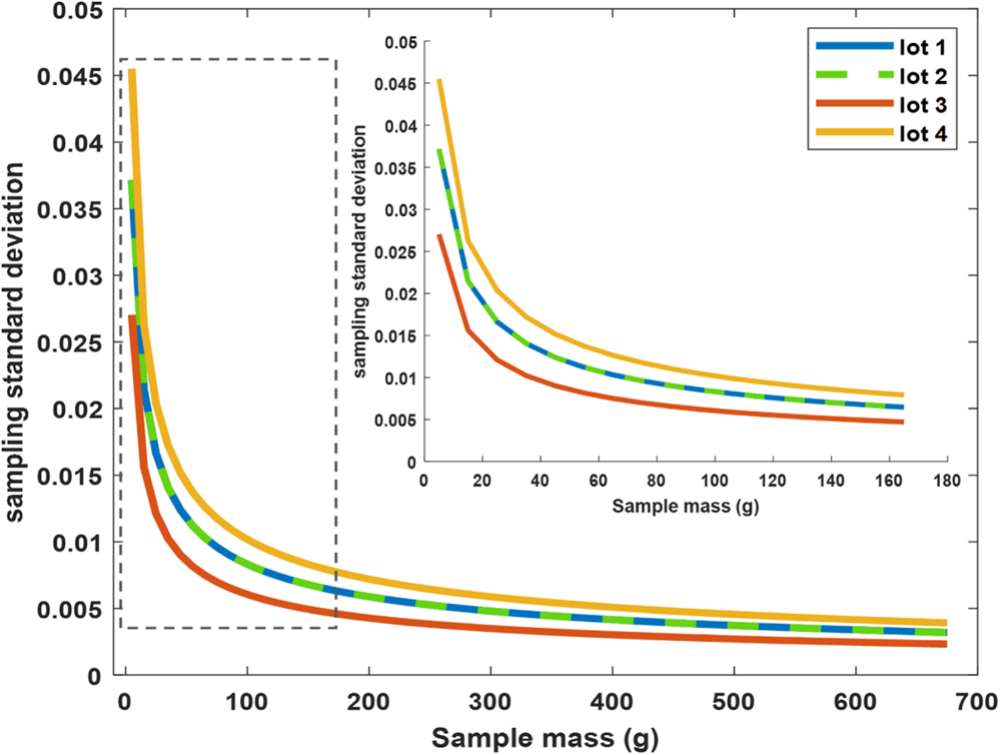
Fundamental sampling error as a function of the sample size for each lot. The inset plot provides a zoom view of sample sizes up to 160g.

As described in Section 2.4.3, the sampling errors of each lot can be evaluated by changing the size of the primary samples. In Figure 4, the sampling standard deviations of different lots are plotted against the sampling mass values for comparison. As all the sampling characteristics of lots 1 and 2 are identical, the sampling standard deviation curves for these two lots completely overlap. For a fixed size of the primary sample, the smallest FSE is observed for lot 3, while the highest value is calculated for lot 4, which is attributed to its larger particle size. Particle size significantly influences the sampling error, with its magnitude being directly proportional to the size cubed (see eq 5).

**Figure 5:**
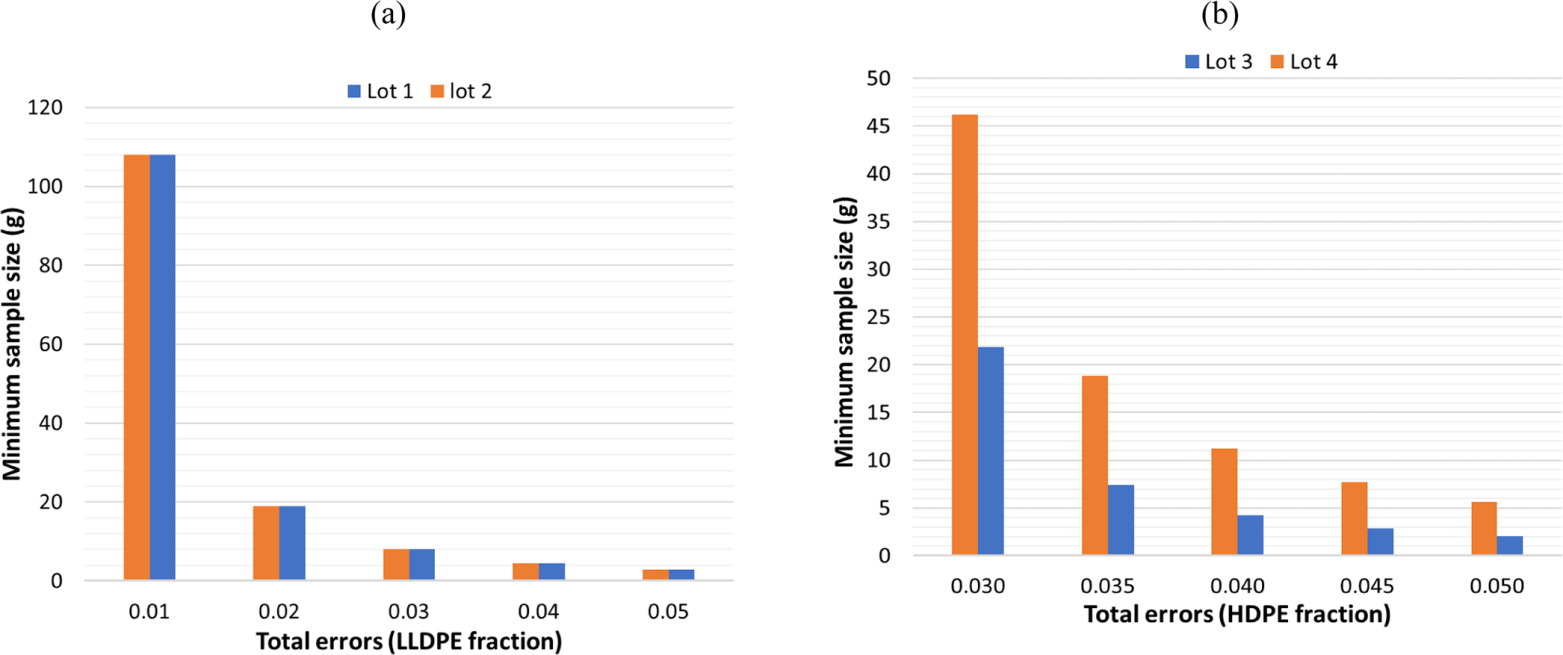
Minimum sample sizes needed to achieve specified levels of total uncertainty for (a) lots 1 and 2, and (b) lots 3 and 4.

The minimum sample size for each lot was calculated based on the maximum allowable total error, which includes both analytical and sampling errors. Using the analytical errors estimated from the PLS analysis, the maximum acceptable sampling errors were calculated, allowing for the determination of the required minimum sample size (see eq 11). Figure 5a,b shows the minimum sample size needed to achieve different levels of total error in each lot. As depicted in Figure 5, the minimum sample size required for LLDPE determination at a maximum allowable total error of 5% (0.05 in fractional terms) is 2.85 g for both lot 1 and lot 2, while for HDPE determination, the required sizes are 2.07 g for lot 3 and 5.68 g for lot 4. These values are determined by the current analytical error of the MADSCAN lab setup. Additionally, Figure 5 illustrates that the minimum sample size increases exponentially as the maximum allowable total uncertainty decreases. For example, to achieve a total error of 0.01 for LLDPE measurements, an analytical sample size of 108 g is required. Similarly, to determine HDPE with a total error of 0.03, an analytical sample size of 46 g is needed from lot 4. This underscores that analyzing only a few milligrams of analytical samples when measuring macro mixes/flakes of polymers is insufficient to meet industry demands or regulatory requirements.

Given that the standard deviation of the analytical error for determining HDPE composition was estimated to be 0.026, achieving total error levels smaller than 0.03 for HDPE determination with the current setup is not possible. As a result, the minimum achievable total error for HDPE is capped at 0.03, while, for LLDPE, it is 0.01 (see Figure 5). This finding highlights the areas where enhanced sensor sensitivity and improved calibration strategies could yield significant benefits. For example, using more sensitive sensors for data acquisition, optimizing sensor design to maximize data accuracy, and refining the calibration range to include more samples with higher fractions of HDPE could improve analytical precision. These insights are particularly valuable for technology providers seeking to advance the accuracy and precision of cross-contamination quantification at the sample scale investigated.

It is important to note that in this study, only a single sample per lot was analyzed, and the current MADSCAN setup operates slower than is typically required for industrial processes. Incorporating additional samples could significantly enhance analytical precision, while reducing the measurement time to around 1–2 h is essential for meeting quality control requirements. To achieve this, future work will focus on increasing the heating rate to 10 °C/min and implementing active cooling at similar rates. An alternative approach under consideration involves a rapid heating phase followed by moderate cooling (e.g., cooling at 5 °C/min, then a second heating at 5 °C/min, with a final rapid cooling phase), thereby maintaining an overall analysis time within the 2-h target while also improving sensitivity. Additionally, employing thinner samples (<5 mm) in future iterations of the MADSCAN setup is expected to provide a more uniform thermal profile and further enhance the precision of the analysis.

The data analysis framework proposed in this study offers a roadmap for technology providers to evaluate sensor sensitivity, calibration strategies, and overall data acquisition techniques, which are advancements critical to the circular economy. Moreover, these results underscore the fundamental importance of applying sampling theory in scenarios where accurately quantifying cross-contamination is essential for reducing quality loss. This approach bridges theoretical principles and practical measurement challenges, ensuring more representative and reliable analyses across diverse industrial applications.

## 4 Conclusions

The transition of the plastics industry to a fully circular model depends on the ability to produce high-quality products from recycled plastics. This necessitates robust quality control measures capable of overcoming existing limitations in accurately quantifying impurities and polymer compositions, particularly in large recyclate batches. This study presents a data analysis framework to estimate cross-contamination levels in recyclate batches, incorporating the minimum sample size required to achieve an industry-allowable total error of ≤ 5% based on the theory of sampling. The calculations account for both sampling and analytical measurement uncertainties.

Additionally, this work introduces MADSCAN, a scale-free technique based on differential scanning calorimetry that enhances impurity detection and facilitates the analysis of large sample sizes needed to account for the heterogeneity in large recyclate batches. This technique overcomes the limitations of current thermal methods, which can analyze only small sample amounts extracted from large volumes of recycled materials.

Analysis of samples from various plastic waste streams demonstrated the effectiveness of the MADSCAN technique, when coupled with PLS analysis, in accurately determining the composition of HDPE and LLDPE mixtures. The minimum sample sizes for quantifying cross-contamination in these mixtures were found to be 2.85 g for LLDPE and 2.07 g for HDPE at a maximum allowable total error of 0.05. These results underscore the shortcomings of current thermal approaches, which can analyze only samples weighing a few milligrams.

Determining the required sample sizes is crucial for guiding industry stakeholders in developing future quality control regulations and for assisting technology providers, such as MADSCAN, in refining sample size requirements for their final developments.
